# Genetic Basis and Physiological Effects of Lipid A Hydroxylation in *Pseudomonas aeruginosa* PAO1

**DOI:** 10.3390/pathogens8040291

**Published:** 2019-12-10

**Authors:** Alessandra Lo Sciuto, Matteo Cervoni, Roberta Stefanelli, Maria Concetta Spinnato, Alessandra Di Giamberardino, Carmine Mancone, Francesco Imperi

**Affiliations:** 1 Department of Science, Roma Tre University, 00146 Roma, Italymariaconcetta.spinnato@uniroma3.it (M.C.S.); 2 Department of Biology and Biotechnology Charles Darwin, Sapienza University of Rome, Laboratory affiliated to Istituto Pasteur Italia–Fondazione Cenci Bolognetti, 00185 Roma, Italy; roberta.stefanelli@uniroma1.it; 3 Department of Molecular Medicine, Sapienza University of Rome, 00185 Roma, Italy; alessandra.digiamberardino@uniroma1.it (A.D.G.); carmine.mancone@uniroma1.it (C.M.)

**Keywords:** biofilm, *Galleria mellonella*, hydroxylation, infection, lipid A, LpxO1, LpxO2, resistance, virulence

## Abstract

Modifications of the lipid A moiety of lipopolysaccharide influence the physicochemical properties of the outer membrane of Gram-negative bacteria. Some bacteria produce lipid A with a single hydroxylated secondary acyl chain. This hydroxylation is catalyzed by the dioxygenase LpxO, and is important for resistance to cationic antimicrobial peptides (e.g., polymyxins), survival in human blood, and pathogenicity in animal models. The lipid A of the human pathogen *Pseudomonas aeruginosa* can be hydroxylated in both secondary acyl chains, but the genetic basis and physiological role of these hydroxylations are still unknown. Through the generation of single and double deletion mutants in the *lpxO1* and *lpxO2* homologs of *P. aeruginosa* PAO1 and lipid A analysis by mass spectrometry, we demonstrate that both LpxO1 and LpxO2 are responsible for lipid A hydroxylation, likely acting on different secondary acyl chains. Lipid A hydroxylation does not appear to affect in vitro growth, cell wall stability, and resistance to human blood or antibiotics in *P. aeruginosa*. In contrast, it is required for infectivity in the *Galleria mellonella* infection model, without relevantly affecting in vivo persistence. Overall, these findings suggest a role for lipid A hydroxylation in *P. aeruginosa* virulence that could not be directly related to outer membrane integrity.

## 1. Introduction

The cell envelope of diderm (Gram-negative) bacteria consists of two concentric membranes, the cytoplasmic (or inner) and the outer membrane, separated by the periplasmic space. The inner membrane is a typical phospholipids bilayer, while the outer membrane of most diderm bacteria is composed of glycerophospholipids and lipopolysaccharide (LPS) in the inner and outer leaflet, respectively [1]. LPS is a negatively charged glycolipid that, in the presence of divalent cations, forms a tightly packed layer which provides an effective permeability barrier against harmful agents [2].

*Pseudomonas aeruginosa* is a Gram-negative opportunistic pathogen mostly dreaded for its high antibiotic resistance and for its ability to cause severe infections in immunocompromised patients. It also represents the main cause of chronic lung infection in individuals suffering from cystic fibrosis, bronchiectasis, or chronic obstructive pulmonary disease [3]. *P. aeruginosa* LPS consists of a hydrophobic lipid A moiety, that anchors the molecule to the outer membrane, a conserved core oligosaccharide and a distal O-antigen polysaccharide, that can be present in two chemically distinct types, historically referred to as A-band and B-band O polysaccharides. B-band O polysaccharides can vary widely among strains and represent the basis of *P. aeruginosa* O-serotyping [4]. Both genetics and chemical evidences have confirmed LPS as an essential component of the *P. aeruginosa* cell envelope [5,6,7]. 

**Figure 1:**
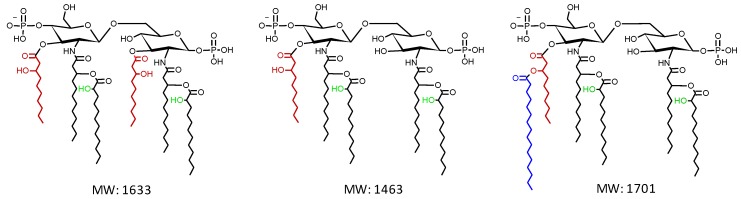
Chemical structure and molecular weight (MW) of the lipid A species most commonly found in *P. aeruginosa,* i.e., the hexa-acylated lipid A (left), the penta-acylated lipid A (middle), and the penta-acylated lipid A with a secondary C16 (palmitoyl) chain (right). The primary and secondary C12 acyl chains are in black, the primary C10 chains are in red, and the palmitoyl chain is in blue. The predicted hydroxyl groups of the secondary C12 acyl chains are highlighted in green. Chemical structures were drawn with the ACD/ChemSketch software, which also calculated the MW.

*P. aeruginosa* lipid A consists of an N- and O-acylated di-glucosamine bisphosphate backbone (Figure 1). While the basic lipid A structure is quite conserved among strains, some heterogeneity is observed in the number of primary acyl groups and the number and nature of secondary acyl groups [4,8]. *P. aeruginosa* lipid A is mostly present with five or six fatty acid substituents, including two N-linked 12:0 (3-OH) groups at positions 2 and 2′ (primary C12 or lauryl chains), one or two O-linked 10:0 (3-OH) groups at positions 3 and/or 3′ (primary C10 or decanoyl chains), and two 12:0 groups O-linked to the primary C12 chains (secondary C12 chains). In most strains, a fraction of lipid A contains an additional secondary 16:0 (palmitoyl) group linked to the primary C10 chain at position 3′ (Figure 1) [4,8].

*P. aeruginosa* lipid A from different strains and culture conditions also show a variable degree of hydroxylation of the secondary C12 chains [4,8,9,10]. In most of the Gram-negative bacteria that produce lipid A with hydroxylated secondary acyl chains, the incorporation of the hydroxyl group in lipid A fatty acids is catalyzed by the alpha-ketoglutarate-dependent dioxygenase LpxO, first characterized in *Salmonella* [11,12]. In *Klebsiella pneumoniae* and *Acinetobacter baumannii*, lipid A hydroxylation increases resistance to cationic antimicrobial peptides (e.g., polymyxins) and is required for full virulence, as demonstrated in the insect *Galleria mellonella* infection model [13,14]. Lipid A hydroxylation was also found to be important for *Burkholderia pseudomallei* replication within macrophages [15], and for *K. pneumoniae* persistence in the lung of experimentally infected mice [16]. 

Interestingly, while all LpxO-proficient Gram-negative bacteria investigated so far have a single hydroxylated secondary acyl chain in lipid A [12,13,14,15], the lipid A of *P. aeruginosa* can be hydroxylated in both secondary C12 chains, and two *lpxO* orthologs have been identified in *P. aeruginosa* genomes, named *lpxO1* and *lpxO2* [4,8,10] (www.pseudomonas.com). However, the involvement of these two genes in lipid A hydroxylation, as well as the role of this lipid A modification in *P. aeruginosa* physiology, has not been experimentally investigated thus far.

In this work, we used reverse genetics and mass spectrometry to confirm that both *lpxO1* and *lpxO2* are responsible for lipid A hydroxylation, likely acting on different C12 chains. We also demonstrate that, differently from other Gram-negative bacteria, lipid A hydroxylation has no relevant effects on *P. aeruginosa* PAO1 growth, cell envelope integrity, and antibiotic and human blood resistance, although it is required for pathogenicity in the *G. mellonella* model of acute infection.

## 2. Materials and Methods

### 2.1. Bacterial Strains and Growth Conditions

Bacterial strains used in this study are listed in Table S1. Bacteria were cultured in Lysogeny Broth, Lennox formulation (Acumedia) for genetic manipulation, while growth, biofilm, and antibiotic resistance assays were performed in Mueller–Hinton broth (MH; Difco) or M9 minimal medium [17] supplemented with 50 μM FeCl_3_ and 20 mM succinate as the carbon source. Planktonic growth assays were performed in 96-well microtiter plates (200 µL of medium in each well) at 22, 30, or 37 °C and the optical density at 600 nm (OD_600_) of bacterial cultures was measured in a Tecan Spark 10M microtiter reader. When required, antibiotics were added for the selection of recombinant strains on agar plates at the following concentration for *E. coli* (the concentration used for *P. aeruginosa* is shown between brackets): nalidixic acid 20 µg/mL, chloramphenicol 30 µg/mL (350 µg/mL).

### 2.2. Generation of Deletion Mutants

Unmarked in-frame deletion mutants in *lpxO1* and *lpxO2* genes were obtained using the *sacB*-based suicide plasmid pDM4 [18] (Table S1). *E. coli* was used for recombinant DNA manipulations. The constructs for mutagenesis were generated by directional cloning into pBluescript II (Stratagene) two DNA fragments of ca. 500 bp, encompassing the regions upstream and downstream of the gene to be deleted. All DNA fragments were amplified by PCR with Q5 Hot Start High-Fidelity DNA Polymerase (New England Biolabs) using the genomic DNA of *P. aeruginosa* PAO1 as the template. Primers and restriction enzymes used for cloning are described in Table S2. After DNA sequencing checks, the upstream and downstream fragments were excised from pBluescript II and sub-cloned into pDM4, yielding pDM4Δ*lpxO1* and pDM4Δ*lpxO2* (Table S1). The pDM4 derivatives were conjugally transferred from *E. coli* S17.1λ*pir* into *P. aeruginosa*, and in-frame deletion mutations were obtained by recombination and sucrose-based selection as described [19]. Gene deletions in *P. aeruginosa* were verified by PCR and DNA sequencing.

### 2.3. Generation of Plasmids

The pME*lpxO1* and pME*lpxO2* constructs were generated by PCR amplification and directional cloning of the DNA fragment encompassing the coding sequence of each gene into the isopropyl-1-thio-ß-D-galactopyranoside (IPTG) inducible shuttle vector pME6032 [20], using the primers and restriction enzymes reported in Table S2. The constructs were verified by DNA sequencing and introduced in *P. aeruginosa* strains by transformation using chemically competent cells.

### 2.4. Lipid A Analysis

Lipid A was extracted from bacterial cell pellets using the ammonium hydroxide-isobutyric acid-based procedure as previously described [10,21]. Briefly, cells from 2 mL of early stationary phase cultures in MH were collected by centrifugation and resuspended in 400 μL of 70% (v/v) isobutyric acid and 1 M ammonium hydroxide (5:3). After 1-h incubation at 100 °C and centrifugation at 2000× *g* for 15 min, supernatants were added to 400 μL of endotoxin-free water, frozen at −80 °C, and lyophilized in a centrifugal vacuum concentrator. The resultant pellets were washed with 1 mL methanol, and lipid A was extracted using 100 μL of chloroform, methanol, and water (3:1:0.25). After centrifugation at 2000× *g* for 15 min, 2 μL of the supernatants were mixed with 2 μL of 10 mg/mL norharmane matrix in chloroform:methanol (2:1), and 0.5 μL of the mixtures were spotted on a matrix-assisted laser desorption-time of flight (MALDI-TOF) plate (5800 MALDI TOF/TOF Analyzer, Sciex, Ontario, Canada). Samples were analyzed in the negative-ion mode with reflectron mode. Calibration was performed using default calibration originated by five standard spots. Spectral data were analyzed with the 4000 Series Explorer software Version 4.1.0 (Sciex, Ontario, Canada), and used to estimate lipid A forms based on their predicted structures and molecular weights [10].

### 2.5. Detergent Sensitivity Assay

Sensitivity to the lytic effect of sodium dodecyl sulphate (SDS) was assessed by determining the turbidity (OD_600_) of bacterial cells from late-exponential cultures resuspended in saline after incubation at room temperature for 5 minutes in the presence of increasing concentrations of SDS (0–5%, w/v) [22].

### 2.6. Antibiotics Sensitivity Assays

Sensitivity to many different antibiotics was assessed by the Kirby–Bauer disc diffusion test. Bacterial cell suspensions in saline were normalized at 0.5 McFarland Standard and swabbed onto MH agar plates, using disks containing ciprofloxacin (5 µg), novobiocin (30 µg), rifampicin (5 µg), erythromycin (15 µg), streptomycin (10 µg), tobramycin (10 µg), imipenem (10 µg), and colistin (10 µg) (Becton Dickinson). Growth inhibition halos were measured after 24 h of growth at 22, 30, or 37 °C.

Colistin sensitivity was also assessed with minimum inhibitory concentration (MIC) assay using the broth microdilution method. Briefly, strains were cultured in MH at 37 °C for 8 h, and then refreshed at 5 × 10^5^ cells/ml in the same medium in the presence of increasing concentrations of colistin (up to 16 μg/mL). MIC was defined as the lowest concentration of antibiotics for which no visible growth was observed after 24 h at 37 °C. Each strain was tested in at least three independent experiments.

### 2.7. Biofilm Assay

Bacterial attachment and biofilm formation were evaluated through the microtiter plate biofilm assay. Bacteria were refreshed in MH from mid-exponential cultures at an OD_600_ = 0.01 and aliquoted in 96-well polystyrene plates (180 µL per well). Plates were incubated for 24 h at 22, 30, or 37 °C under static conditions. The wells were washed several times with distilled water, the attached cells were stained with 220 µL of 0.1% crystal violet at room temperature for 15 min, and the wells were washed again several times with distilled water to remove unbound dye. Biofilm-bound crystal violet was eluted with 200 µL of 30% acetic acid at room temperature for 15 min and, for each well, 100 μL of the resulting solution were aliquoted in a new microtiter plate. The released crystal violet was measured as OD_595_ in a Tecan Spark 10M microtiter reader. Four biological replicates with four wells each were performed for each strain.

### 2.8. Whole Blood Killing Assay

Whole blood killing assay was performed as described [13], with few modifications. Briefly, bacteria were grown in MH until the late-exponential phase, harvested by centrifugation and resuspended in saline at OD_600_ = 1. Three hundred microliters of fresh human blood, pooled from three healthy donors, were mixed with 100 μL of bacterial suspensions, and incubated at 37 °C and 220 rpm. Serial dilutions in saline were plated onto MH agar plate at time 0 and after 1-h incubation with human blood to obtain viable counts. Human serum was obtained from healthy volunteers who gave their written informed consent to the study. The research project was approved by the review board of the Pasteur Institute-Cenci Bolognetti Foundation, Sapienza University of Rome (Rome, Italy).

### 2.9. Galleria Mellonella Infection and Persistence Assays

*G. mellonella* larvae were purchased from the Serpens breeding (Paliano, Italy; www.bigserpens.com) and used within one day of shipment. *P. aeruginosa* strains were grown in MH until mid-exponential phase of growth, and serial ten- or three-fold dilutions of bacterial cell suspensions in saline were injected into *G. mellonella* larvae as described [23]. Infected larvae were incubated at 30 °C for up to 3 days to monitor mortality. Ten larvae were infected with each infecting dose in three independent assays. Kaplan–Meier survival curves, lethal doses 90% (LD_90_), and R^2^ values were obtained using GraphPad Prism as described [24].

*P. aeruginosa* persistence in *G. mellonella* larvae was assessed as described [22], with few modifications. Briefly, larvae were infected with about 10^6^ bacterial cells and, after 2, 4, or 8 h of incubation at 30 °C, cut with a razor blade to recover the hemolymph. Ten-fold serial dilutions of the hemolymph were plated on MH agar supplemented with 100 μg/mL ampicillin (to which *P. aeruginosa* is intrinsically resistant) to determine the percentage of viable cells with respect to the infecting dose.

*P. aeruginosa* persistence in *G. mellonella* hemolymph was also assessed *ex vivo*. About 10^6^ bacterial cells were inoculated in the hemolymph collected and pooled from ten larvae and, after 2, 4, or 8 h of incubation at 30 °C, the samples were serially diluted in saline and plated on MH agar supplemented with 100 μg/mL ampicillin.

### 2.10. Statistical Analyses

Statistical analysis was performed with GraphPad Instat, using One-Way Analysis of Variance (ANOVA) followed by Tukey-Kramer multiple comparison test. Statistical analysis of survival curves of *G. mellonella* larvae or bacterial persistence in larvae was performed with GraphPad Prism, using the Log-rank (Mantel–Cox) test or the Kruskal–Wallis test followed by Dunn’s multiple comparison test, respectively.

## 3. Results and Discussion

### 3.1. Both LpxO1 and LpxO2 are Responsible for Lipid A Hydroxylation

**Figure 2:**
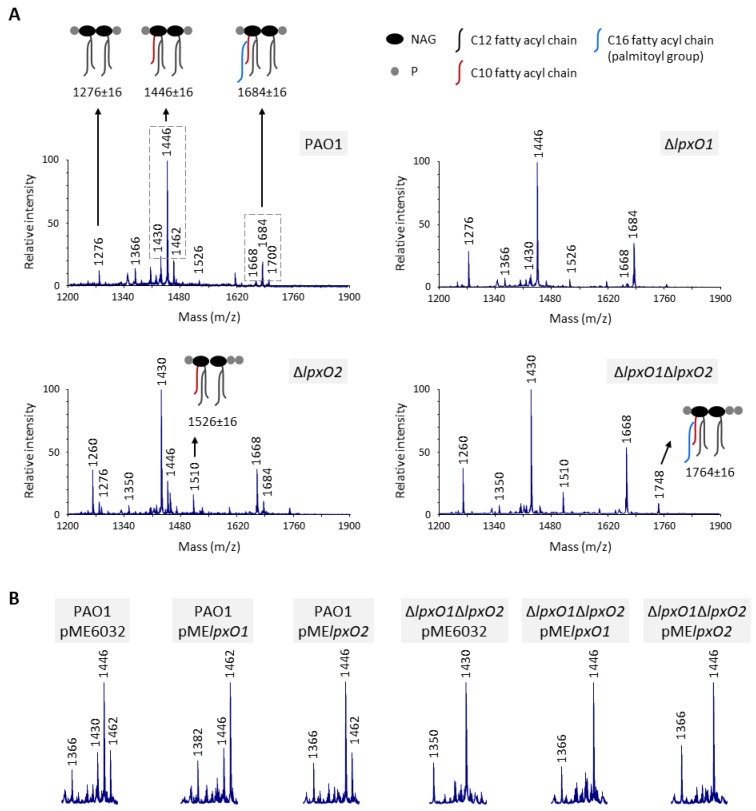
Role of LpxO1 and LpxO2 in lipid A hydroxylation. (**A**) MALDI-TOF analysis of lipid A extracted from *P. aeruginosa* wild type (PAO1) and *lpxO1* and/or *lpxO2* mutant cells cultured in Mueller–Hinton broth (MH) at 37 °C until the late-exponential growth phase. Spectra were obtained in the ion negative mode, thus m/z values correspond to (molecular mass–1)/1, and are representative of three biological replicates. Relevant lipid A forms are shown with cartoons: m/z = 1276, diphosphorylated tetra-acylated lipid A; m/z = 1446, diphosphorylated penta-acylated lipid A; m/z = 1526, triphosphorylated penta-acylated lipid A; m/z = 1684, diphosphorylated penta-acylated lipid A with a palmitoyl group; and m/z = 1764, triphosphorylated penta-acylated lipid A with a palmitoyl group. m/z ± 16 indicates lipid A forms that vary for the presence/absence of a hydroxyl group to each secondary C12 acyl chain. (**B**) Details of the MALDI-TOF spectra of lipid A extracted from PAO1 or the Δ*lpxO1*Δ*lpxO2* mutant carrying constructs for LpxO1 or LpxO2 overexpression (pME*lpxO1* or pME*lpxO2*) or the empty vector pME6032, cultured in the presence of 0.5 mM isopropyl-1-thio-ß-D-galactopyranoside (IPTG). The images highlight the diphosphorylated penta-acylated lipid A species at m/z = 1430, 1446, and 1462 (non-, mono-, and dihydroxylated, respectively), while the corresponding full-length spectra are shown in Figure S2.

In order to verify the involvement of *lpxO* homologs in the hydroxylation of secondary C12 chains of lipid A in *P. aeruginosa*, single and double in-frame deletion mutants in *lpxO1* (PA4512) and/or *lpxO2* (PA0936) were generated in the reference laboratory strain PAO1. Lipid A was extracted from mutant and wild type cells and analyzed by MALDI-TOF mass spectrometry. In line with previous evidence [10], the main lipid A form of the wild type strain PAO1 is the diphosphorylated penta-acylated lipid A, with a fraction of this molecule modified by the addition of palmitoyl group to the C10 acyl chain (Figure 2A). For both these lipid A variants, the MALDI spectra showed three distinct peaks with m/z differences of 16, due to the presence/absence of a hydroxyl group in the secondary C12 acyl chains [4], although the peaks corresponding to the monohydroxylated forms (m/z = 1446 and 1684) were much more abundant (Figure 2A). Notably, a reduction in the lipid A hydroxylation state was observed in all *lpxO* mutants. In particular, the Δ*lpxO1* mutant lost the peaks corresponding to dihydroxylated lipid A, but the monohydroxylated forms remained predominant. In contrast, the lipid A of the Δ*lpxO2* mutant was mainly present in the non-hydroxylated state (m/z = 1430 and 1668), although monohydroxylated peaks were also detected. Finally, only non-hydroxylated lipid A was observed in the Δ*lpxO1*Δ*lpxO2* double mutant (Figure 2A). Besides the hydroxylation state, a slight increase was observed in the relative intensity of peaks corresponding to tetra-acylated lipid A in all mutants and of triphosphorylated lipid A forms in Δ*lpxO2* strains only (Figure 2A). While these lipid A forms were previously observed in *P. aeruginosa* [25,26,27], whether they have any effects on the properties of the LPS layer is not known. Ectopic expression of *lpxO2* in the Δ*lpxO2* mutant from an IPTG-inducible promoter restored the lipid A hydroxylation pattern of the wild type (Figure S1). In contrast, IPTG-induced expression of *lpxO1* in the Δ*lpxO1* mutant significantly increased the relative intensity of dihydroxylated lipid A peaks (Figure S1), suggesting that the minor effect of *lpxO1* deletion on lipid A hydroxylation (Figure 2A) is likely due to poor expression and/or activity of the endogenous *lpxO1* gene under the growth conditions tested in this work. 

Altogether, these data demonstrate that both LpxO homologs of *P. aeruginosa* can hydroxylate the secondary C12 chains of lipid A, although LpxO2 appears to be responsible for most of lipid A hydroxylation, at least in the reference strain PAO1 and/or in our experimental setting. Moreover, the complete lack of dihydroxylated lipid A forms in both *lpxO1* and *lpxO2* single mutants, which instead retain mono-hydroxylated species (Figure 2A), strongly suggests that each LpxO enzyme hydroxylates a specific C12 chain. This hypothesis was confirmed by assessing the effect of individual LpxO1 or LpxO2 overexpression in wild type and Δ*lpxO1*Δ*lpxO2* backgrounds. In the wild type strain, LpxO1 overexpression strongly increased the relative intensity of dihydroxylated lipid A peaks, while LpxO2 overexpression only led to the loss of non-hydroxylated forms (Figure 2B and Figure S2). This confirms that, in our experimental setting, LpxO2 is the most active enzyme in PAO1, while LpxO1 is likely poorly expressed from its endogenous promoter. On the other hand, overexpression of either LpxO1 or LpxO2 in the Δ*lpxO1*Δ*lpxO2* double mutant restored monohydroxylation of lipid A, but dihydroxylated species were not observed for any overexpressed enzyme (Figure 2B and Figure S2). This finding further supports the hypothesis that the two LpxO proteins of *P. aeruginosa* have specificity for different C12 chains. Unfortunately, our attempts to distinguish the position of the hydroxyl groups by MALDI-TOF/TOF tandem mass spectrometry analysis of monohydroxylated lipid A extracted from the Δ*lpxO1*Δ*lpxO2* mutant individually overexpressing each LpxO homolog did not clarify this issue (data not shown). Further experiments and/or different genetics and biochemical approaches will therefore be required to elucidate the specificity of the two *P. aeruginosa* LpxO enzymes.

### 3.2. Lipid A Hydroxylation Does Not Affect in vitro Growth, Cell Envelope Integrity and Resistance

**Figure 3:**
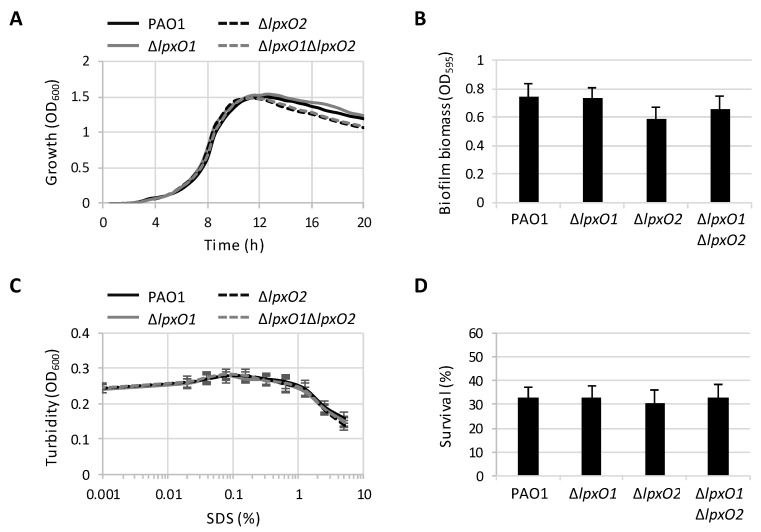
Effect of lipid A hydroxylation on *P. aeruginosa* planktonic and biofilm growth cell envelope integrity and human blood resistance. (**A**) Planktonic growth (OD_600_) of the wild type PAO1 and Δ*lpxO1*, Δ*lpxO2,* and Δ*lpxO1*Δ*lpxO2* deletion mutants in MH at 37 °C. Growth curves are representative of three independent experiments performed in triplicate. (**B**) Biofilm formation in 96-well polystyrene microtiter plates of PAO1 and *lpxO* mutants after 24 h at 37 °C under static conditions. Values are the mean (±SD) from four independent assays. (**C**) Lytic effect of sodium dodecyl sulphate (SDS) at different concentrations (0%–5%), measured as a decrease in cell suspension turbidity (OD_600_), on wild type and *lpxO* mutant cells. Values are the mean (±SD) from three independent experiments performed in duplicate. (**D**) Survival of PAO1 and *lpxO* mutant cells in whole human blood after 1-h incubation at 37 °C. Values are the mean (±SD) from three independent experiments, and are expressed as percentage of viable cells with respect to the initial inoculum. Differences were not statistically significant (*P* > 0.05; ANOVA).

Since the parental strain and the different *lpxO* mutants showed various degrees of lipid A hydroxylation (Figure 2), we used these strains to assess whether lipid A hydroxylation can affect *P. aeruginosa* physiology. First, we compared planktonic growth among the wild type and *lpxO* mutant strains. Growth kinetics and yields were almost identical, both in complex (Figure 3A) and minimal media (Figure S3). In addition, only minor, non-significant differences were observed in biofilm formation between wild type and *lpxO* mutants (Figure 3B), indicating that lipid A hydroxylation is not required for both planktonic and biofilm growth in *P. aeruginosa*.

We then assessed the role of lipid A hydroxylation in antibiotic resistance, by comparing the activity of different antibiotics with different chemical properties and mechanisms of action against the wild type and *lpxO* mutant strains through the Kirby–Bauer disc diffusion assay. As reported in Table 1, we did not observe any variation in drug resistance in any mutant and for any antibiotic. Since the poor agar diffusion of colistin hampers the detection of minor differences in resistance through the disk diffusion test [28], and considering that lipid A hydroxylation was found to affect polymyxin resistance in many different bacteria, such as *Vibrio cholerae*, *K. pneumonia,* and *A. baumannii* [13,14,29], we confirmed that all strains have the same colistin MIC using the broth microdilution method (Table 1). These results rule out any specific effect of the hydroxylation state of lipid A on antibiotic resistance, as well as any general role in the permeability barrier of the *P. aeruginosa* outer membrane. This is in line with the finding that all strains showed a comparable resistance profile to the lytic effect of the detergent SDS (Figure 3C), which has been previously used to reveal defects in outer membrane stability [7,10,22], thus implying that the integrity of the cell envelope of *P. aeruginosa* PAO1 is not affected by changes in the lipid A hydroxylation levels.

Whole blood killing assay has been recently used to highlight the contribution of lipid A hydroxylation to resistance to professional phagocytes in *A. baumannii* and *K. pneumoniae* [13,14]. We therefore sought to verify *ex vivo* whether LpxO1 and/or LpxO2 affect *P. aeruginosa* PAO1 survival in human blood. Once again, none of the *lpxO* mutants showed significant differences in survival in whole human blood with respect to their parental strain (Figure 3D), indicating that hydroxylation in the lipid A secondary chains does not contribute to blood resistance in *P. aeruginosa*.

Notably, *lpxO1* was found to be slightly up-regulated (about two-fold) at 25 °C as compared to 37 °C by transcriptomic analysis [30], and a possible correlation between a specific single nucleotide polymorphism (SNP) in *lpxO1* and biofilm formation at 22 °C has been recently proposed through genome-wide association analysis [31]. To investigate any possible temperature-dependent effects of LpxO enzymes, we performed growth, biofilm, and antibiotic resistance assays also at 22 and 30 °C. As shown in Figure S4, we did not observe any relevant differences between the wild type and *lpxO* mutants under both conditions. While this further confirms that lipid A hydroxylation is not required for growth, biofilm formation, and for proper permeability barrier of the outer membrane, we cannot rule out that it may have more subtle effects on *P. aeruginosa* physiology that cannot be highlighted by our assays.

### 3.3. Lipid A Hydroxylation Contributes to P. aeruginosa Virulence in an Insect Model of Infection

Finally, to gain insights into the possible role of lipid A hydroxylation during *P. aeruginosa* PAO1 infection, we assessed the pathogenicity of lipid A hydroxylation-defective mutants in the well-established *G. mellonella* model of infection [32], which represents a convenient and easy-to-handle infection model to preliminary screen the infectivity of *P. aeruginosa* mutants [23].

**Figure 4:**
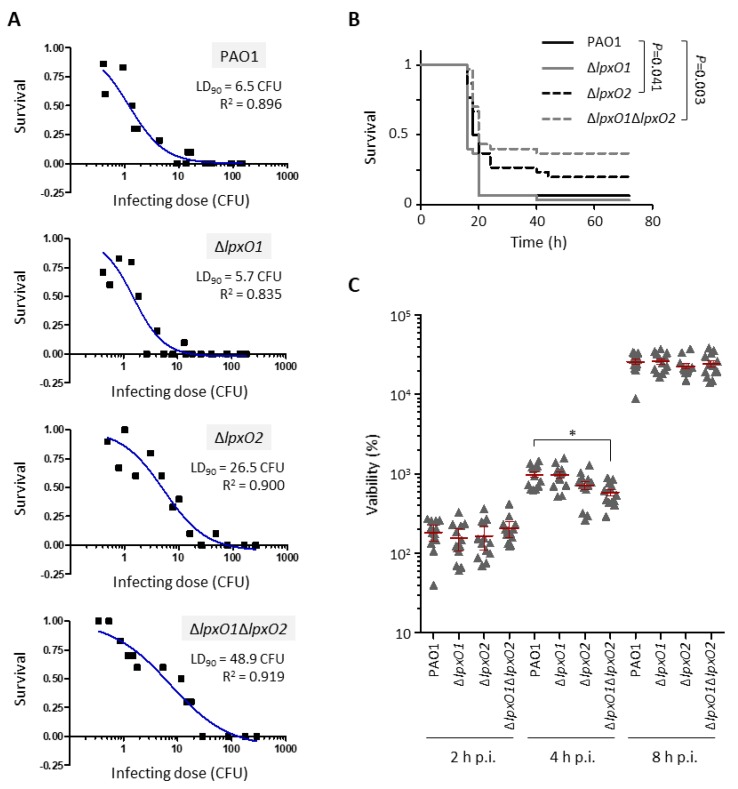
Effect of lipid A hydroxylation on *P. aeruginosa* infectivity in *G. mellonella*. (**A**) Dose-dependent survival curves of *G. mellonella* larvae infected with different doses of the wild type PAO1, the Δ*lpxO1*, the Δ*lpxO2,* or the Δ*lpxO1*Δ*lpxO2* deletion mutant. Ten larvae were infected with each infecting dose in three independent experiments. LD_90_ and R^2^ values were determined by GraphPad Prism and are reported in the figure. Abbreviation: CFU, colony-forming units. (**B**) Kaplan–Meier survival curves of *G. mellonella* larvae infected with PAO1 (14.9 ± 1.6 CFU), Δ*lpxO1* (15.4 ± 2.9 CFU), Δ*lpxO2* (14.0 ± 3.4 CFU), or Δ*lpxO1*Δ*lpxO2* (14.8 ± 3.2 CFU). Thirty larvae were infected with each strain in three independent experiments. Only statistically significant differences are shown in the figure, according to the Log-rank (Mantel–Cox) test. (**C**) Viability of PAO1, Δ*lpxO1*, Δ*lpxO2,* or Δ*lpxO1*Δ*lpxO2* cells in *G. mellonella* larvae at 2, 4, and 8 h post-infection (p.i.), reported as a percentage with respect to the infecting dose. Means with 95% confidence intervals are shown in red. For each time point, thirteen larvae per strain were infected in three independent assays. The asterisk indicates a statistically significant difference (*P* < 0.05) with respect to the wild type at the same time point (Kruskal–Wallis).

Differently from all the in vitro results, mutants impaired in lipid A hydroxylation were found to be significantly attenuated in pathogenicity in *G. mellonella* larvae. Indeed, we observed a four- and almost eight-fold increase in the LD_90_ of the Δ*lpxO2* and Δ*lpxO1*Δ*lpxO2* mutants with respect to the wild type, respectively (Figure 4A). The reduced infectivity of these two mutants was confirmed by Kaplan–Maier survival curves obtained with a definite infecting dose (about 15 bacterial cells) (Figure 4B). In contrast, both the LD_90_ values and the timing of killing of the Δ*lpxO1* mutant were basically identical to those of the parental strain (Figure 4A and Figure 4B), indirectly implying that LpxO2 is the main enzyme involved in lipid A hydroxylation during *G. mellonella* infection, in agreement with in vitro observations (Figure 2). 

In order to assess whether the reduced infectivity of mutants defective in lipid A hydroxylation could be due to impaired persistence of bacterial cells in vivo during the infection, *G. mellonella* larvae were infected with a high infecting dose (about 10^6^ CFUs) and the number of *P. aeruginosa* cells in the hemolymph was determined at 2, 4, and 8 h post-infection. We only observed a slight delay in growth in *G. mellonella* larvae for the Δ*lpxO2* mutant and the Δ*lpxO1*Δ*lpxO2* double mutant, although the difference with the wild type was statistically significant only for Δ*lpxO1*Δ*lpxO2* at 4 h (Figure 4C). Notably, no significant differences between wild type and any *lpxO* mutant were observed when bacterial cell viability was assessed *ex vivo* in the hemolymph extracted from *G. mellonella* larvae (Figure S5). This result, together with those obtained from in vitro assays, suggests that LpxO enzyme(s) and lipid A hydroxylation could contribute to *P. aeruginosa* in vivo growth and virulence in *G. mellonella* larvae in a manner that does not directly depend on the integrity of the cell envelope and/or resistance to the antimicrobial peptides of the *G. mellonella* hemolymph.

## 4. Conclusions

Several studies on different Gram-negative pathogens have highlighted the importance of lipid A modifications in the adaptation of the outer membrane to harsh environments and to the host during infections [33,34,35,36]. In *P. aeruginosa,* the enzymes responsible for the addition/removal of phosphate, palmitoyl, hydroxydecanoyl, and lauryl groups to/from lipid A, as well as their effects on outer membrane remodeling and bacterial physiology, have been characterized [9,37,38,39]. In contrast, the role of hydroxylation of secondary lipid A fatty acid chains was not previously examined in this species, although *P. aeruginosa* is the only Gram-negative pathogen reported so far with two hydroxylated secondary fatty acids in lipid A. Here, we demonstrated that the two *lpxO* homologs of *P. aeruginosa* are both functional and hydroxylate different secondary chains, even if *lpxO2* accounts for the majority of lipid A hydroxylation, at least in the reference strain PAO1 and under the in vitro and in vivo conditions investigated in our study. Interestingly, we observed that, differently from other bacterial pathogens, lipid A hydroxylation does not affect the integrity and permeability barrier of the *P. aeruginosa* outer membrane, and accordingly is not required for in vitro growth, ex vivo blood resistance, and persistence in *G. mellonella* hemolymph. Nevertheless, we found that mutants impaired in lipid A hydroxylation are significantly attenuated in virulence in the *G. mellonella* model of acute infection. This suggests that lipid A hydroxylation could also play some role in bacterial virulence other than (or additional to) its previously reported effect on outer membrane integrity and resistance. This work paves the way for future studies aimed at confirming the importance of lipid A hydroxylation in other *P. aeruginosa* infection models, and/or at unravelling the molecular basis of lipid A hydroxylation-dependent effect on *P. aeruginosa* virulence.

## Figures and Tables

**Table 1:** Antibiotic susceptibility of *P. aeruginosa lpxO* mutants by the Kirby–Bauer disk diffusion and/or minimum inhibitory concentration (MIC) assays ^1^.

Strain	Inhibition Halo (mm) ^2^								MIC (μg/ml) ^3^
	Cip	Novo	Rif	Ery	Sm	Tob	Imp	Ct	Ct
PAO1	30.6 (±0.5)	6.0 (±0.0)	6.0 (±0.0)	6.0 (±0.0)	12.9 (±1.3)	22.2 (±1.7)	25.0 (±1.4)	12.5 (±0.5)	0.5
Δ*lpxO1*	30.8 (±1.6)	6.0 (±0.0)	6.0 (±0.0)	6.0 (±0.0)	12.6 (±0.7)	23.2 (±3.1)	24.2 (±0.5)	12.5 (±0.5)	0.5
Δ*lpxO2*	30.9 (±1.1)	6.0 (±0.0)	6.0 (±0.0)	6.0 (±0.0)	11.9 (±1.5)	22.0 (±0.9)	25.2 (±0.8)	12.5 (±0.5)	0.5
Δ*lpxO1*Δ*lpxO2*	30.8 (±2.2)	6.0 (±0.0)	6.0 (±0.0)	6.0 (±0.0)	12.4 (±1.8)	23.0 (±2.8)	24.4 (±1.2)	12.5 (±0.5)	0.5

^1^ Abbreviations: Cip, ciprofloxacin; Novo, novobiocin; Rif, rifampicin; Ery, erythromycin; Sm, streptomycin; Tob, tobramycin; Ipm, imipenem; Ct, colistin. ^2^ Values are the mean (±SD) of four independent assays. ^3^ Values are the mode of six independent assays.

## Supplementary Materials

The following are available online at https://www.mdpi.com/2076-0817/8/4/291/s1, Figure S1: MALDI-TOF lipid A spectra of Δ*lpxO1* and Δ*lpxO2* mutants carrying the complementing plasmid pME*lpxO1* or pME*lpxO2*, Figure S2: MALDI-TOF lipid A spectra of the PAO1 and the Δ*lpxO1*Δ*lpxO2* mutant overexpressing LpxO1 or LpxO2, Figure S3: Bacterial growth curves in minimal medium, Figure S4: Growth, biofilm formation and antibiotic sensitivity profile at 22 and 30 °C, Figure S5: Bacterial viability in the *G. mellonella* hemolymph *ex vivo*, Table S1: Bacterial strains and plasmids used in this study, Table S2: Primers used in this study.
