# Supplementary material for: Genetic Basis and Physiological Effects of Lipid A Hydroxylation in Pseudomonas aeruginosa PAO1
Source: Pathogens. 2019 Dec 10;8(4):291. doi: 10.3390/pathogens8040291 (PMC6963906; doi:10.3390/pathogens8040291)
Supplement: Supplementary file 1 [file pathogens-08-00291-s001.zip › pathogens-646971-supplementary/Figure S1-S5.pdf]

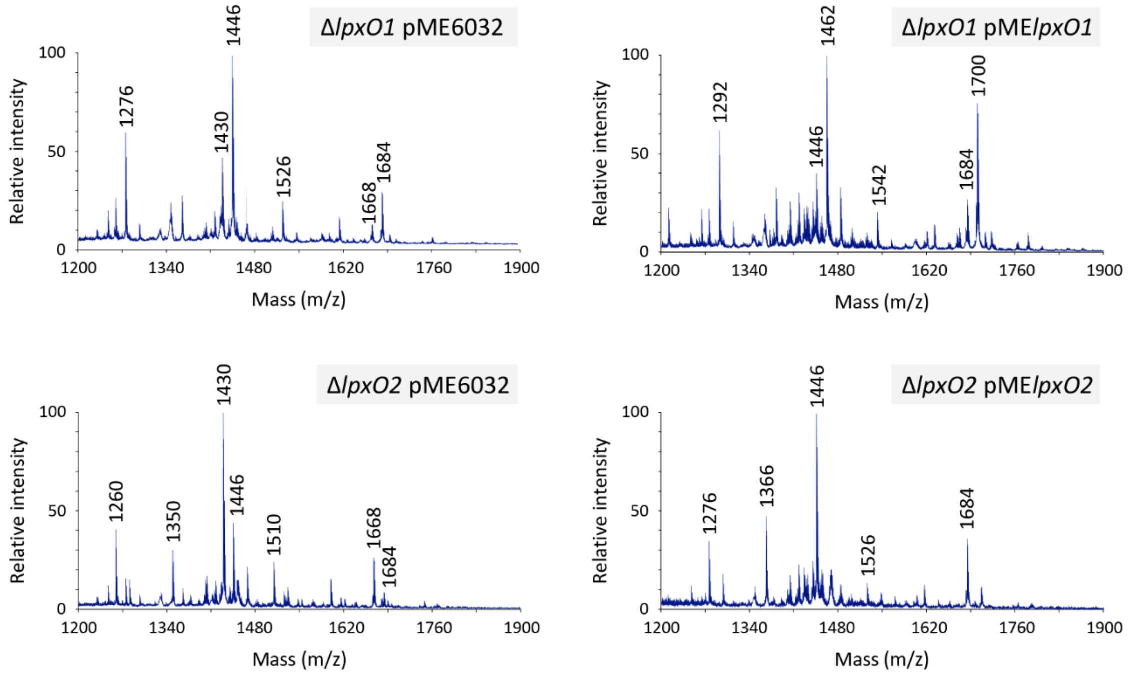

Figure S1. MALDI-TOF analysis of lipid A extracted from the *P. aeruginosa* Δ*lpxO1* and Δ*lpxO2* mutants carrying the complementing plasmid pME*lpxO1* or pME*lpxO2* or the empty vector pME6032 as control. Strains were cultured at 37°C in MH supplemented with 0.5 mM IPTG to induce *lpxO1* or *lpxO2* expression. Spectra are representative of two biological replicates.

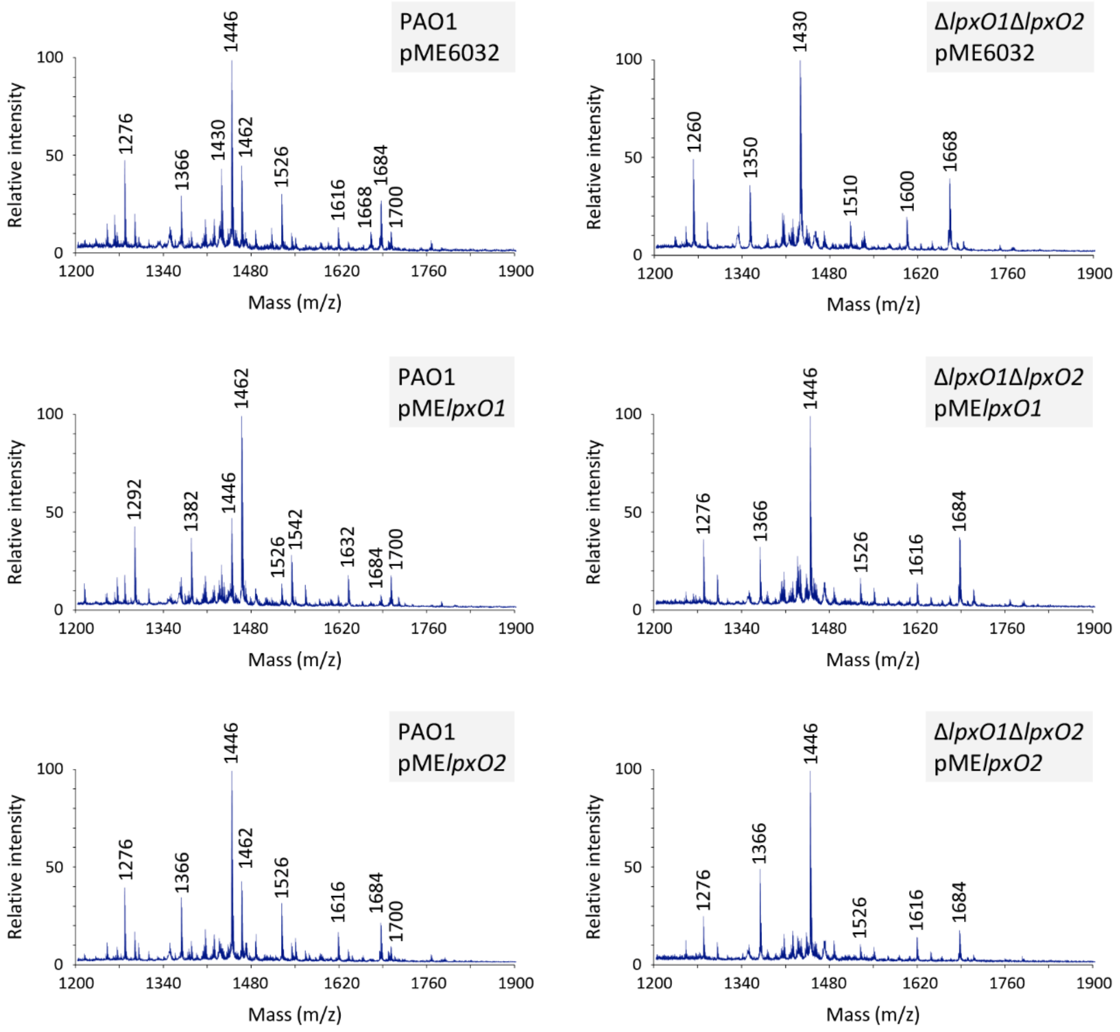

Figure S2. MALDI-TOF analysis of lipid A extracted from the wild type strain PAO1 and the Δ*lpxO1*Δ*lpxO2* double mutant carrying the complementing plasmid pME*lpxO1* or pME*lpxO2* or the empty vector pME6032 as control. Strains were cultured at 37°C in MH supplemented with 0.5 mM IPTG to overexpress *lpxO1* or *lpxO2*. Spectra are representative of two biological replicates.

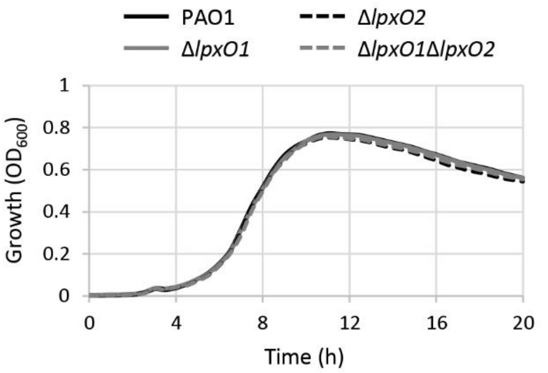

Figure S3. Growth curves in minimal medium. Planktonic growth (OD_{600}) of the wild type PAO1 and Δ*lpxO1*, Δ*lpxO2* and Δ*lpxO1*Δ*lpxO2* deletion mutants at 37°C in M9 supplemented with 50 μM $FeCl_3$ and succinate as carbon source. Growth curves are representative of three independent experiments performed in triplicate.

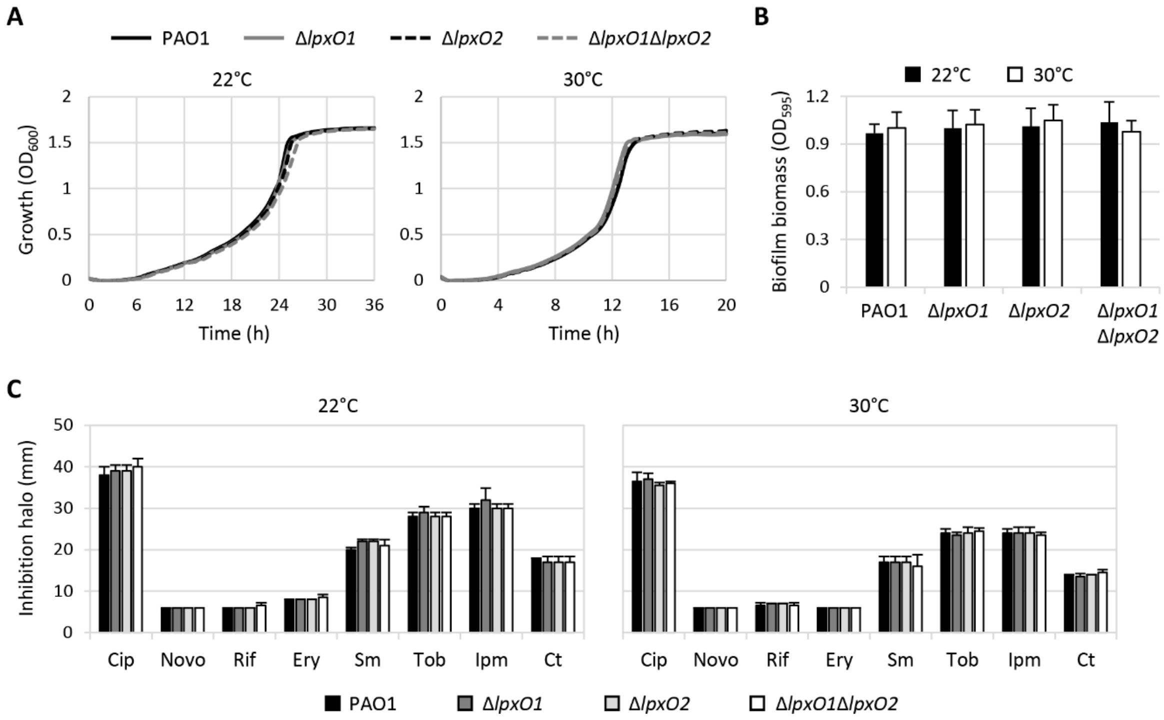

Figure S4. Effect of lipid A hydroxylation on planktonic and biofilm growth and on the antibiotic sensitivity profile of *P. aeruginosa* at 22 and 30°C. (A) Planktonic growth (OD_{600}) of the wild type PAO1 and *lpxO* mutants in MH at 22 or 30°C. Growth curves are representative of three independent experiments performed in triplicate. (B) Biofilm formation in 96-well polystyrene microtiter plates of PAO1 and *lpxO* mutants after 24 h at 22 or 30°C under static conditions. Values are the mean (±SD) from four biological replicates. (C) Antibiotic susceptibility of PAO1 and *lpxO* mutants at 22 or 30°C, determined by the Kirby-Bauer disk diffusion assay. Values are the mean (±SD) of three independent assays. Antibiotic abbreviations: Cip, ciprofloxacin; Novo, novobiocin; Rif, rifampicin; Ery, erythromycin; Sm, streptomycin; Tob, tobramycin; Ipm, imipenem; Ct, colistin.

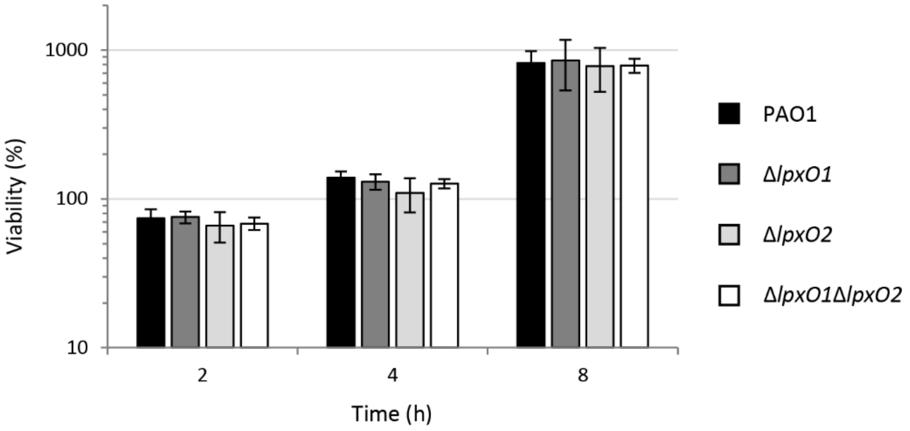

Figure S5. Viability over time of the wild type PAO1 and Δ*lpxO1*, Δ*lpxO2* and Δ*lpxO1*Δ*lpxO2* deletion mutants at 30°C in *G. mellonella* hemolymph *ex vivo*. Values are expressed as percentage with respect to time 0 (100%), and represent the mean (±SD) of three independent experiments. In each experiment, the hemolymph was collected from ten larvae and pooled.
