# Supplementary material for: Genetic Basis and Physiological Effects of Lipid A Hydroxylation in Pseudomonas aeruginosa PAO1
Source: Pathogens. 2019 Dec 10;8(4):291. doi: 10.3390/pathogens8040291 (PMC6963906; doi:10.3390/pathogens8040291)
Supplement: Supplementary file 1 [file pathogens-08-00291-s001.zip › pathogens-646971-supplementary/Table S1.pdf]

**Table S1.** Bacterial strains and plasmids used in this study.

| Strain or plasmid | Genotype and/or relevant characteristics | Reference or source |
|---|---|---|
| ***P. aeruginosa*** | | |
| PAO1 (ATCC15692) | Prototroph | American type culture collection |
| Δ*lpxO1* | PAO1 derivative with an in-frame deletion in the *lpxO1* (PA4512) coding sequence | This work |
| Δ*lpxO2* | PAO1 derivative with an in-frame deletion in the *lpxO2* (PA0936) coding sequence | This work |
| Δ*lpxO1*Δ*lpxO2* | Δ*lpxO1* derivative with an in-frame deletion in the *lpxO2* coding sequence | This work |
| ***E. coli*** | | |
| S17.1λ*pir* | *thi pro hsdRhsdM*$^{+}$*recA RP4-2-Tc::Mu-Km::Tn7 λpir,* $Sm^R$ | Simon *et al.* 1983 |
| **Plasmid** | | |
| pBluescript II (pBS) | Sequencing vector; ColE1 replicon; $Ap^R$ | Stratagene |
| pBS *lpxO1*↑ *lpxO1*↓ | pBS derivative containing the regions upstream and downstream of the *lpxO1* coding sequence | This work |
| pBS *lpxO2*↑ *lpxO2*↓ | pBS derivative containing the regions upstream and downstream of the *lpxO1* coding sequence | This work |
| pDM4 | Suicide vector; *sacBR*, *oriR6K*; $Cm^R$ | Milton *et al.* 1996 |
| pDM4Δ*lpxO1* | pDM4 derivative for *lpxO1* in-frame deletion, generated by sub-cloning the *lpxO1*↑ *lpxO1*↓ fragment of pBS *lpxO1*↑ *lpxO1*↓into pDM4, $Cm^R$ | This work |
| pDM4Δ*lpxO2* | pDM4 derivative for *lpxO2* in-frame deletion, generated by sub-cloning the *lpxO2*↑ *lpxO2*↓ fragment of pBS *lpxO2*↑ *lpxO2*↓into pDM4, $Cm^R$ | This work |
| pME6032 | Shuttle vector for IPTG inducible expression in *P. aeruginosa*, $Tc^R$ | Heeb *et al.* 2002 |
| pME*lpxO1* | pME6032 derivative carrying the *lpxO1* coding sequence under the control of the IPTG-inducible promoter | This work |
| pME*lpxO2* | pME6032 derivative carrying the *lpxO2* coding sequence under the control of the IPTG-inducible promoter | This work |

Reference non included in the main text:

Simon, R.; Priefer, U; Pühler, A. A broad host range mobilization system for *in vivo* genetic engineering: transposon mutagenesis in Gram negative bacteria. *Bio/Technology.* **1983**, *1, 784-790.*
