# Supplementary material for: Genetic Basis and Physiological Effects of Lipid A Hydroxylation in Pseudomonas aeruginosa PAO1
Source: Pathogens. 2019 Dec 10;8(4):291. doi: 10.3390/pathogens8040291 (PMC6963906; doi:10.3390/pathogens8040291)
Supplement: Supplementary file 1 [file pathogens-08-00291-s001.zip › pathogens-646971-supplementary/Table S2.pdf]

Table S2. Primers used in this study.[a]

**Primer name**	**Sequence (5'→3')**[b]	**Restriction site**	**Application**
*lpxO1*_mut_UP_FW	ccgctcgaGTACCGAGGGAACGGCAG	XhoI	Generation of pDM4Δ*lpxO1*
*lpxO1*_mut_UP_RV	cgggatcCAGCGCATAGGCCGCCAG	BamHI	
*lpxO1*_mut_DOWN_FW	cgggatCCGAACAACGCAGGCGAC	BamHI	
*lpxO1*_mut_DOWN_FW	gctctAGATCTATGTCCAGGACCGC	XbaI	
*lpxO2*_mut_UP_FW	ccgctcgaGTCGACCTGGTTCTTGTGC	XhoI	Generation of pDM4Δ*lpxO2*
*lpxO2*_mut_UP_RV	cgggatCCAGTTGGCGCAGGAAGG	BamHI	
*lpxO2*_mut_DOWN_FW	cgggatcCGGTCGCCATTCGCGGC	BamHI	
*lpxO2*_mut_DOWN_FW	gctctagAGAGGGTGACGAGGGTGC	XbaI	
*lpxO1*_pME6032_FW	cggaatTCCTCTTTTGCACCGACGAC	EcoRI	Generation of pME*lpxO1*
*lpxO1*_pME6032_RV	ccgctcGAGCTATCGGGGCCTTGC	XhoI	
*lpxO2*_pME6032_FW	catgccatGGCCCCTGATATCGAAGGC	NcoI	Generation of pME*lpxO2*
*lpxO2*_pME6032_RV	ccgctcgaGAATCAGCCGAAGATCCAAC	XhoI	
M13FW	GTTTTCCCAGTCACGAC		DNA sequencing
M13RV	CAGGAAACAGCTATGAC		DNA sequencing

[a] PCRs were performed using the genomic DNA of *P. aeruginosa* PAO1 as the template.
[b] The restriction site used for cloning is underlined in the primer sequence.
